# Artificial Intelligence-based Psychotherapy Practices

**DOI:** 10.1192/j.eurpsy.2025.220

**Published:** 2025-08-26

**Authors:** F. H. Kraxner, EPA Section of Psychotherapy

**Affiliations:** Center of Psychiatry and Psychotherapy, Hospital of Affoltern, Zurich, Affoltern, Switzerland

## Abstract

**Abstract:**

Artificial Intelligence (AI) is revolutionizing the field of psychotherapy by introducing innovative, accessible, and efficient methods for mental health care. AI-based psychotherapy practices leverage machine learning algorithms, natural language processing (NLP), and predictive analytics to provide personalized therapeutic interventions, support mental health practitioners, and enhance patient outcomes. These systems, including virtual therapists and chatbot platforms, are designed to simulate human empathy, analyze user inputs, and deliver evidence-based therapeutic techniques such as cognitive-behavioral therapy (CBT).

Moreover, AI systems assist clinicians by offering diagnostic support, monitoring patient progress through behavioral data analysis, and optimizing treatment plans. The integration of AI into psychotherapy practices has shown promise in addressing barriers such as stigma, geographic limitations, and therapist shortages. However, ethical challenges related to data privacy, algorithmic bias, and the therapeutic efficacy of AI-driven interactions remain significant concerns. This abstract explores the potential, applications, and limitations of AI-based psychotherapy, emphasizing the need for rigorous research and ethical considerations to ensure its effective and responsible integration into mental health care.

**Disclosure of Interest:**

None Declared

